# Paradoxical Roles of Leucine-Rich α_2_-Glycoprotein-1 in Cell Death and Survival Modulated by Transforming Growth Factor-Beta 1 and Cytochrome *c*

**DOI:** 10.3389/fcell.2021.744908

**Published:** 2021-10-08

**Authors:** Ronald Jemmerson

**Affiliations:** Department of Microbiology and Immunology, University of Minnesota, Minneapolis, MN, United States

**Keywords:** LRG1, TGF-β1, cytochrome *c*, apoptosis, cell death, cell survival

## Abstract

Leucine-rich α_2_-glycoprotein-1 (LRG1) has been shown to impact both apoptosis and cell survival, pleiotropic effects similar to one of its known ligands, transforming growth factor-beta 1 (TGF-β1). Recent studies have given insight into the TGF-β1 signaling pathways involved in LRG1-mediated death versus survival signaling, i.e., canonical or non-canonical. Interaction of LRG1 with another ligand, extracellular cytochrome *c* (Cyt *c*), promotes cell survival, at least for lymphocytes. LRG1 has been shown to bind Cyt *c* with high affinity, higher than it binds TGF-β1, making it sensitive to small changes in the level of extracellular Cyt *c* within a microenvironment that may arise from cell death. Evidence is presented here that LRG1 can bind TGF-β1 and Cyt *c* simultaneously, raising the possibility that the ternary complex may present a signaling module with the net effect of signaling, cell death versus survival, determined by the relative extent to which the LRG1 binding sites are occupied by these two ligands. A possible role for LRG1 should be considered in studies where extracellular effects of TGF-β1 and Cyt *c* have been observed in media supplemented with LRG1-containing serum.

## Introduction

Leucine-rich α_2_-glycoprotein-1 (LRG1) is a significant component of blood, as it is secreted by the liver, and is expressed to a lesser extent in other tissues (NCBI gene ID 116844). It was the first member of the large family of proteins containing leucine-rich repeats (LRRs) to be identified (Takahashi et al., 1985). LRG1 has been implicated in a variety of cancers (Andersen et al., 2010; Ladd et al., 2012; Liu et al., 2012), inflammation (Bini et al., 1996; Weivoda et al., 2008), pathogenic angiogenesis (Wang et al., 2013), lymphocyte differentiation (Urushima et al., 2017), and neutrophil function (Druhan et al., 2017). Included among its pleiotropic effects are apoptosis induction (Takemoto et al., 2015; Jin et al., 2019) and cell survival (Codina et al., 2010; Zhou et al., 2017; Xie et al., 2019; Jemmerson et al., 2021), but the molecular basis for this latter distinction has not been defined.

**FIGURE 1:**
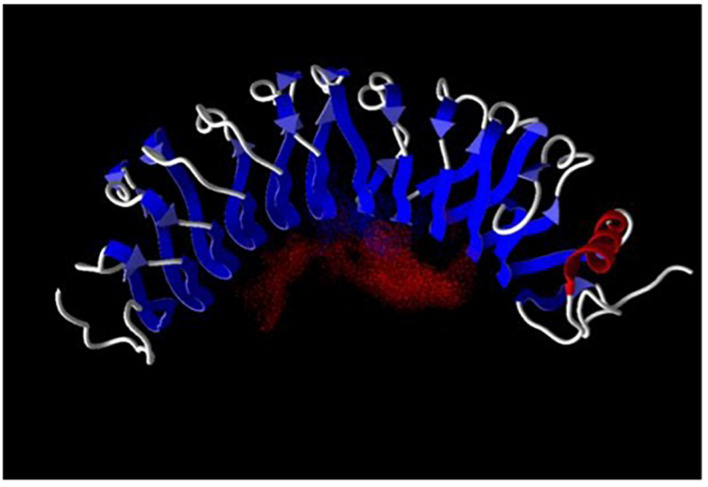
Leucine-rich a_2_-glycoprotein-1 was modeled employing the computer program Molegro (University of Illinois, Urbana-Champaign). Red dots indicate the negative electric field within the ligand binding pocket. Ligand binding-site prediction is based on structural data for a number of other LRR proteins as there are no structural data available for LRG1. The LRRs are shown in blue and α-helix in red.

The three-dimensional structures of a large number of the LRR proteins have been determined (Bella et al., 2008). The repetitive leucine-rich motif in these proteins assumes a curved structure with a concave surface providing a platform for protein–protein interactions (Kobe and Deisenhofer, 1995; Kobe and Kajava, 2001) as exemplified by the model of LRG1 shown in Figure 1. Most ligands of LRR proteins bind within the pocket created by the near horseshoe-shaped polypeptide (Bella et al., 2008). These ligands extend through the pocket similar to a thumb in a doughnut hole as shown, for example, in the crystal structures of ribonuclease A bound to its LRR inhibitor (Kobe and Deisenhofer, 1995) and LRR-containing, G protein-coupled receptor 4 (Lgr4) in complex with R-Spondin-1 (Xu et al., 2013). Recently, synthetic LRR proteins constructed using a consensus LRR sequence were shown to bind simultaneously two muramyl dipeptide ligands with different affinities (Kim et al., 2021). A ternary complex of R-Spondin-1, Lgr5, and ring finger protein 43 has been modeled, further indicating that binding of more than one ligand to LRR-proteins is possible (Chen et al., 2013). This may be facilitated by flexibility in LRRs as the radius of curvature has been shown to increase slightly when a ligand is bound (Kobe and Deisenhofer, 1995).

The negatively charged field in the pocket of LRG1, shown by the red dots in Figure 1, makes it suitable for binding cationic proteins such as cytochrome *c* (Cyt *c*) and transforming growth factor-beta 1 (TGF-β1). The pI of Cyt *c* is 9.6 and of TGF-β1 is 8.6. The molecular weights of LRG1, TGF-β1, and Cyt *c* are 50, 25, and 12.5 kD, respectively. In 2002 TGF-β1 became the first ligand for LRG1 to be identified. In a solid-phase radioimmunoassay TGF-β1 was found to bind a previously unknown protein that was later identified as the mouse homolog of human LRG1 (Saito et al., 2002). More recently TGF-β1 was examined for binding human LRG1 by surface plasmon resonance with LRG1 attached to the sensor chip. The affinity (*K*_*D*_) was calculated to be 2.32 × 10^–6^ M (Takemoto et al., 2015). To my knowledge the stoichiometry for TGF-β1 binding LRG1 has not been determined.

Cytochrome *c* was discovered as a ligand for LRG1 in 2006 when my research group was attempting to quantify Cyt *c* in serum employing a sandwich enzyme-linked immunosorbent assay and observed that a component in serum inhibited Cyt *c* detection in this assay (Cummings et al., 2006). Purification of the inhibitor and analysis by mass spectrometry identified it as LRG1. The specific binding of Cyt *c* to LRG1 was confirmed later by other researchers who employed surface plasmon resonance and, with LRG1 bound to the sensor chip as in the study of TGF-β1, the affinity (*K*_*D*_) was calculated to be 1.58 × 10^–13^ M (Shirai et al., 2010). We have reported that the stoichiometry for Cyt *c* binding LRG1 is 1:1 (Weivoda et al., 2008).

The difference in affinities of LRG1 for these two ligands as reported results from an 80-fold faster on-rate for Cyt *c* than for TGF-β1 and a 10^4^- to 10^5^-fold slower off-rate for Cyt *c* than for TGF-β1.

## Transforming Growth Factor-Beta 1 and Cytochrome *c* Can Bind Leucine-Rich α_2_-Glycoprotein-1 Simultaneously

**FIGURE 2:**
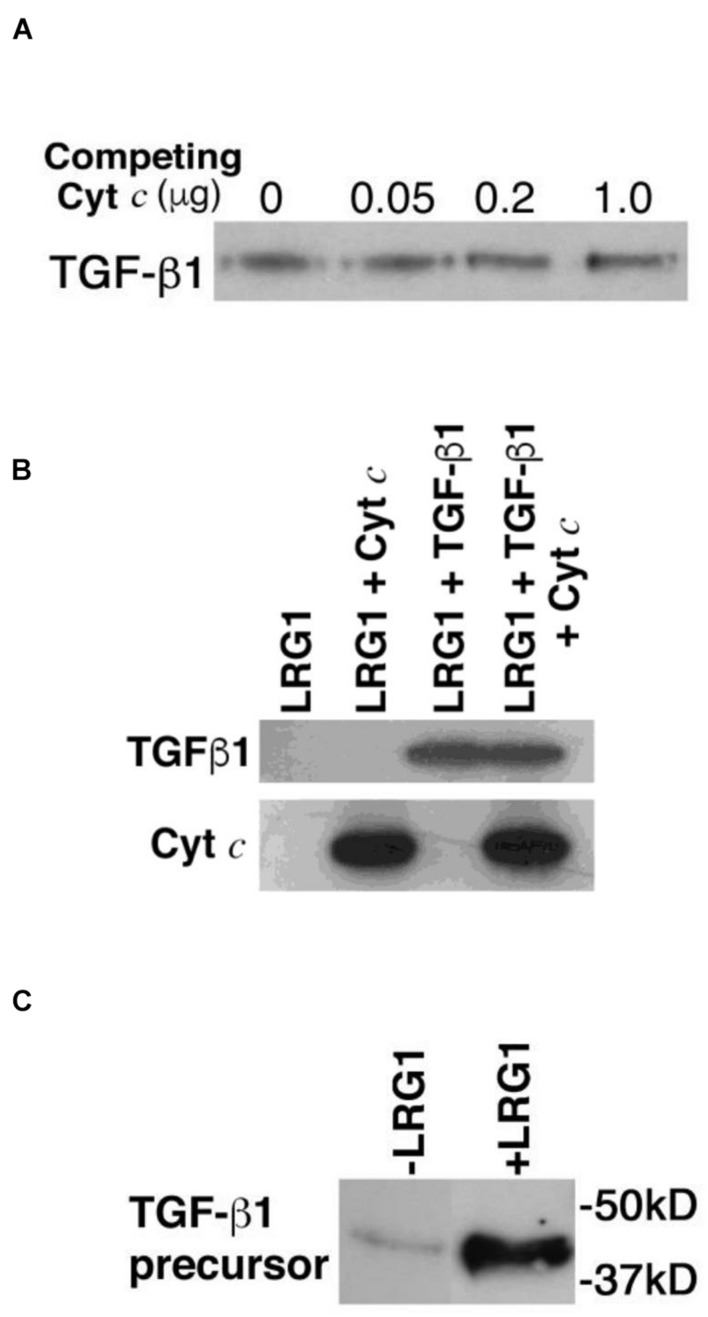
Transforming growth factor-beta 1 and Cyt *c* bind LRG1 simultaneously. **(A)** Cyt *c* does not interfere with TGF-β1 binding to LRG1. Commercially obtained recombinant human TGF-β1 (25 ng; ProSpec, Ness Zima, Israel) was incubated with FLAG-tagged recombinant human LRG1 (100 ng obtained from the supernate of *LRG1* gene-transfected MCF-7 cells and quantified by enzyme-linked immunosorbent assay) along with increasing amounts of horse Cyt *c* (obtained from Sigma Chemical Co., and further purified by ion exchange chromatography using carboxymethyl-Sepharose; note that there is no detectable distinction between human and horse Cyts *c* binding human LRG1). The recombinant LRG1 was immunoprecipitated using anti-FLAG antibody-coupled agarose. Based on the reactivity of antibody-conjugated peroxidase, TGF-β1 was detected in the immunoprecipitates by western blotting (4–20% polyacrylamide gel) employing a commercial rabbit mAb to TGF-β1 (Cell Signaling Technology, Danvers, MA, United States), followed by a secondary antibody specific for rabbit IgG conjugated to horseradish peroxidase. **(B)** Cyt *c* and TGF-β1 are co-immunoprecipitated with LRG1. FLAG-tagged recombinant human LRG1 (1 μg) was incubated with recombinant human TGF-β1 (0.2 μg), horse Cyt *c* (2 μg), or both TGF-β1 and Cyt *c*. In the incubation mixture the molar ratio of LRG1 to TGF-β1 was 2.5-fold (calculated for the TGF-β1 dimer). Cyt *c* was present in an 8-fold molar excess to LRG1 and 20-fold molar excess to TGF-β1. Complexes with LRG1 were immunoprecipitated using anti-FLAG antibody-coupled agarose beads. Bound ligands were visualized by western blotting using mouse mAb 7H8.2C12 to detect Cyt *c* (Liu et al., 1996) and a commercial rabbit mAb to detect TGF-β1 followed by appropriate secondary antibodies coupled to horseradish peroxidase. **(C)** TGF-β1 precursor from human lymphocytes was captured by LRG1 tethered to Cyt *c*-Sepharose beads. Lymphocytes (7 × 10^7^ cells) were solubilized in RIPA buffer containing 1 mM PMSF and the extract was passed through a mini-column containing 0.5 ml Cyt *c*-conjugated Sepharose 4B beads (10 mg protein coupled per ml beads). Non-bound materials were washed through the column using phosphate-buffered saline, pH 7.5 (PBS). Bound molecules were eluted in 1 M acetic acid and freeze dried. Following regeneration of the column in PBS, LRG1 (10 μg, purified from urine of an appendicitis patient) was applied to the column followed by addition of the non-bound material from the previous run. After washing, bound molecules were eluted and freeze dried. TGF-β1 present in the eluates was examined by western blotting. In this case since the starting material was derived from lymphocytes the precursor form of TGF-β1 was observed.

To determine if TGF-β1 and Cyt *c* bind distinct sites on LRG1, first a competition experiment was carried out. Recombinant human TGF-β1 (25 ng) was incubated with increasing concentrations of horse Cyt *c* (0–1 μg) and a constant amount of recombinant FLAG-tagged LRG1 (100 ng), then LRG1 complexes were immunoprecipitated with anti-FLAG antibody-coupled agarose beads. TGF-β1 was visualized in a western blot. Cyt *c*, in vast molar excess of TGF-β1, did not inhibit binding of TGF-β1 to LRG1 (Figure 2A).

Next, to confirm that TGF-β1 and Cyt *c* bind different sites on LRG1 a similar experiment was carried out where Cyt *c* was present in a large molar excess. Both recombinant human TGF-β1 and Cyt *c* were shown to be co-immunoprecipitated with recombinant human LRG1 (Figure 2B).

Simultaneous binding of Cyt *c* and lymphocyte-derived TGF-β1 to human-derived LRG1 was also demonstrated in a different type of experiment. A detergent-extract of lymphocytes was passed through a mini-column of Cyt *c*-coupled Sepharose beads. Any bound material was eluted, then following column regeneration, LRG1, purified from urine of an appendicitis patient, was passed through the column followed by the lymphocyte-extract effluent from the first column run. (Note that LRG1 obtained from urine has the same molecular weight as blood-derived LRG1, 50 kD, and they appear to be identical.) Materials eluted from both column runs were examined for the presence of TGF-β1 by western blotting (Figure 2C). Only a small amount of TGF-β1 was adsorbed to the Cyt *c*-coated agarose during the first column run but more was significantly adsorbed during the second column run. The small amount observed in the first run could be due to remnant LRG1 from the lymphocyte isolation as LRG1 is not known to be produced by lymphocytes. The adsorption of LRG1 to Cyt *c* in the second column run allowed capture of TGF-β1. The higher molecular weight of the lymphocyte-derived TGF-β1 compared to the recombinant form indicates that it was likely the precursor form of TGF-β1 from the lymphocyte extract that was captured in this experiment (Miyazono et al., 1988).

These data, along with previous calculations of affinities as determined by surface plasmon resonance, indicate that LRG1 has two binding sites with vastly different affinities for these two ligands. This finding raises the possibility that the ternary complex of LRG1, TGF-β1, and Cyt *c* may form a signaling module. If so, what is the functional outcome?

## Leucine-Rich α_2_-Glycoprotein-1 Promotes Apoptosis Through Canonical Transforming Growth Factor-Beta 1 Signaling

The first and, perhaps, most thorough analysis to date of cell signaling effected through LRG1 was in a study of pathogenic angiogenesis where LRG1 was found to promote signaling through the TβRII–ALK1–Smad1/5/8 pathway in the presence of TGF-β1 (Wang et al., 2013). Since then TGF-β1 signaling modulated by LRG1 has been implicated in multiple studies including several in which apoptosis induction was observed (Table 1).

**TABLE 1:** Evidence for pro-apoptotic and pro-survival functions of LRG1.

**Effect of LRG1**	**Cells studied**	**Proposed mechanism**	**References**
Pro-apoptotic	Lewis lung (mouse) carcinoma	Extracellular signaling through TGF-β1-Smad2	Takemoto et al., 2015
Pro-apoptotic	Human hepato-carcinoma	Extracellular signaling through TGF-β1-Smad2	Takemoto et al., 2015
Pro-apoptotic	Mouse brain	Extracellular signaling through TGF-β1-Smad1/5	Jin et al., 2019
Pro-apoptotic	Human esophageal squamous carcinoma	Phosphorylation of Smad2/3	Zhang et al., 2020
Pro-survival	Human and mouse lymphocytes	Extracellular signaling through a putative, unidentified receptor requiring LRG1 binding Cyt *c*	Codina et al., 2010
Pro-survival	Colorectal cancer	Increased RUNX1 expression	Zhou et al., 2017
Pro-survival	Pancreatic cancer	Extracellular signaling through EGF receptor/p38	Xie et al., 2019
Pro-survival	HUVEC	Mechanism unknown	Yang et al., 2020
Pro-survival	Glioblastoma	Mechanism unknown	Zhong et al., 2015
Pro-survival	Leukemia	LRG1 downregulation inhibiting JAK–STAT pathway	Xiao and Zhu, 2018
Pro-survival	Breast cancer	Intracellular LRG1 competes with Apaf-1 for binding cytosolic Cyt *c*	Jemmerson et al., 2021

In one study two different cell lines were analyzed for the role of LRG1 in the enhancement of TGF-β1-induced apoptosis (Takemoto et al., 2015). In Lewis lung carcinoma cells mouse LRG1 was overexpressed by transfection causing TGF-β1-induced proliferation to be inhibited in those cells. There was decreased expression of bcl-2 enabling translocation of Cyt *c* from mitochondria and increased activity of several caspases indicating increased apoptosis in the *LRG1*-transfected cells in response to TGF-β1. Western blotting showed increased phosphorylation of Smad2 and Smad1/5/8 demonstrating signaling through a canonical TGF-β1 pathway. Furthermore, the phosphorylation of Smad2 was blocked by an inhibitor of the TGFβRI receptor which also enhanced tumor growth in mice.

Leucine-rich α_2_-glycoprotein-1 was decreased by RNA silencing in the other cell line examined in the study, a human hepatocellular carcinoma. In response to TGF-β1 there was decreased caspase activity indicating less apoptosis in cells expressing lower amounts of LRG1 and a decrease in phosphorylation of Smad2. Human LRG1 was observed in the culture fluid of the parental carcinoma cells leading the investigators to propose that extracellular LRG1 was likely responsible for the TGF-β1-induced apoptosis of the parental cells.

In another study LRG1 was overexpressed in mice using an adenovirus vector to introduce the gene and then the middle cerebral artery was occluded (Jin et al., 2019). This induced apoptosis in brain tissue as shown by a decrease in bcl-2 expression, increased expression of Bax (a promoter of Cyt *c* translocation from mitochondria), and increased caspase 3 activity. Simultaneously, ALK1 expression increased along with phosphorylation of Smad1/5, implicating the same TGF-β1 signaling pathway as in the original pathogenic angiogenesis study.

Apoptosis effected by LRG1 was also shown in a study of human esophageal squamous cell carcinoma by a decrease in bcl-2 and increases in Bax and caspase 3 expression when LRG1 was overexpressed by *LRG1* transfection and opposite levels as a consequence of LRG1 RNA silencing (Zhang et al., 2020). Smad2/3 phosphorylation was examined by western blotting and there was, perhaps, a slight increase in phosphorylation of Smad2/3 in cells with decreased LRG1 but no apparent difference in phosphorylated Smad2/3 between control cells and cells overexpressing LRG1. Taken at face value this finding may implicate a TGF-β signaling pathway involved in LRG1-induced apoptosis in this case.

## A Survival Function for Leucine-Rich α_2_-Glycoprotein-1 With Evidence for a Non-Canonical Transforming Growth Factor-Beta 1 Signaling Pathway

There have been several other studies demonstrating a pro-survival function for LRG1 (Table 1). In some of those studies an attempt was made to identify signaling molecules. Knock-down of LRG1 in colorectal cancer cells by RNA silencing induced apoptosis as shown by a decrease in cell cycling factors and the level of bcl-2, while Bax and cleaved caspase 3 increased. When LRG1 was added to the medium, the expression of the transcription factor RUNX1 increased, cell growth was promoted, and the levels of Bax and cleaved caspase 3 were deceased (Zhou et al., 2017).

In a study of pancreatic cancer, knock down of LRG1 mRNA decreased viability of two cell lines and overexpression of LRG1 by gene transfection increased viability, proliferation, and cell migration (Xie et al., 2019). The phosphorylation of p38 was found to correlate with the level of LRG1 expression. An inhibitor of the epidermal growth factor receptor (EGFR) blocked the LRG1 effect on p38 phosphorylation and, in cells where LRG1 was overexpressed by gene transfection, LRG1 was co-immunoprecipitated with the EGFR.

Knockdown of LRG1 in leukemia cells by RNA silencing resulted in a decrease in cell viability due to apoptosis shown by decreased expression of bcl-2 and an increased level of cleaved caspase 3 (Xiao and Zhu, 2018). The effect appeared to be due to a blockage of the Janus kinase (JAK)/signal transducers and activators of transcription (STAT) pathway as phosphorylated levels of JAK2 and STAT3 were decreased.

These three studies implicate non-canonical TGF-β1 signaling in the pro-survival function of LRG1. RUNX transcription factors are key targets for activation by the TGF family (Ito and Miyazono, 2003) and synergy in cancer has been shown between EGFR and TGF-β1 (Uttamsingh et al., 2008) as well as between JAK/STAT3 and TGF-β1 (Liu et al., 2014). Furthermore, the EGFR and JAK/STAT pathways are connected (Andl et al., 2004). A possible role for Cyt *c* was not examined in these studies.

It is relevant to note that miR-335, the micro RNA that downregulates several genes in TGF-β non-canonical pathways, also downregulates LRG1 (Lynch et al., 2012) and that LRG1 and TGF-βII receptor are coordinately expressed (Li et al., 2007).

## Distinction Between the Effects of Intracellular and Extracellular Leucine-Rich α_2_-Glycoprotein-1

In the studies cited in Table 1 it was not always clear whether the effects of LRG1 resulted from intracellular or extracellular activity. In two studies LRG1 was added to the culture medium to show an effect (Codina et al., 2010; Zhou et al., 2017). In another study where LRG1 was observed in the culture fluid it was inferred that the observed effect was due to extracellular LRG1 (Takemoto et al., 2015).

Most of the studies cited in Table 1 involved manipulation of *LRG1* gene expression. Cells that express LRG1, in particular in low quantities, do not necessarily secrete the protein. For example, LRG1 was not detected in the fluid of MCF-7 breast cancer cell cultures (Jemmerson et al., 2021). However, following transfection of the *LRG1* gene the quantity of LRG1 measured in the medium increased dramatically. If LRG1 is secreted, an effect by extracellular versus intracellular LRG1 could be distinguished employing antibodies that block the function of LRG1 (Wang et al., 2013).

The proposed mechanisms of extracellular and not intracellular LRG1 presented in Table 1 may be inferred by the implication of cell-surface receptors in many of the cases. In only one study cited in Table 1 was intracellular LRG1 shown to be involved in cell survival (Jemmerson et al., 2021). As noted above, MCF-7 cells did not secrete LRG1 in a measurable amount in that study. Furthermore, LRG1 was detected in the cytoplasm and knockdown of LRG1 expression resulted in an increase of Cyt *c* bound to Apaf-1 in cytosolic extracts.

A complication in distinguishing effects due to intracellular versus extracellular LRG1 is that if fetal bovine serum is included in the culture medium, it does contain LRG1 that can impact cell viability (see Figure 4). This issue can be resolved by depleting LRG1 from the medium, such as by adsorption using Cyt *c*-conjugated beads.

## Leucine-Rich α_2_-Glycoprotein-1 Promotes Lymphocyte Survival When Bound to Cytochrome *c*

The role of intracellular Cyt *c* in the induction of apoptosis is well documented (Liu et al., 1996). We and others have reported that within a couple of hours following activation of apoptosis intact Cyt *c* begins to be released from apoptotic cells *in vitro* and is immediately released from cells in response to excessive heat (Renz et al., 2001; Ahlemeyer et al., 2002; Jemmerson et al., 2002). In addition, it has been reported that Cyt *c* is localized to secretory granules in the rat pancreas and anterior pituitary suggesting the possibility that extracellular Cyt *c* may play a role in healthy tissues (Soltys et al., 2001). Cyt *c* has been detected at low levels in the blood of healthy individuals (generally, in the low ng/ml range) (Renz et al., 2001; Pullerits et al., 2005; Eleftheriadis et al., 2016) and is elevated in the blood of individuals where extensive apoptosis is indicated such as a consequence of chemotherapy or in various pathologies including liver disease, kidney injury, myocardial infarction, and brain damage, among others (Renz et al., 2001; Eleftheriadis et al., 2016).

Extracellular Cyt *c* has been shown to cause apoptosis in cultured lymphocytes (Codina et al., 2010), to enhance apoptosis induced by staurosporine in primary neuronal cultures (Ahlemeyer et al., 2002), and to induce apoptosis in cultured BV-2 microglial cells (Gouveia et al., 2017). It is not known how extracellular Cyt *c* induces apoptosis. However, Cyt *c* is well known to bind anionic phospholipids (Tuominen et al., 2002) and could perturb the plasma membrane without necessarily binding a signaling receptor (Bergstrom et al., 2013). Also, when bound to phospholipids Cyt *c* undergoes a conformational change with loss of the sixth ligation to the heme enabling it to assume peroxidase activity (Hannibal et al., 2016). Resulting oxidation reactions at the cell membrane could induce apoptosis (Redza-Dutordoir and Averill-Bates, 2016).

**FIGURE 3:**
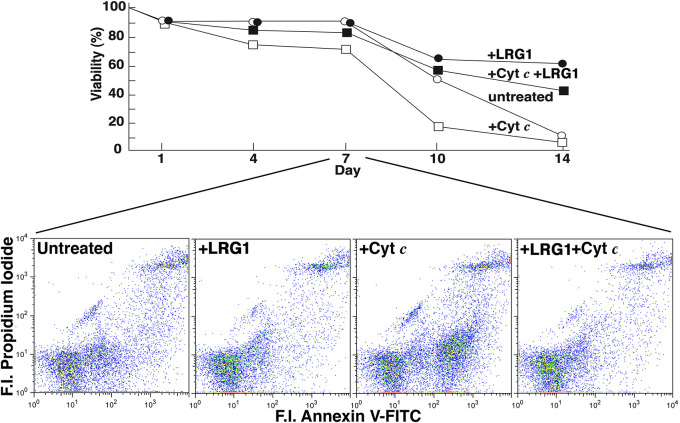
Leucine-rich α_2_-glycoprotein-1 protects human lymphocytes from cell death induced by extracellular Cyt *c*. Cells were cultured in 10% fetal bovine serum-containing Dulbecco’s modified Eagle’s medium, depleted of LRG1 by adsorption on Cyt *c*-Sepharose 4B and supplemented with antibiotics. The lymphocytes (total blood mononuclear cells) were obtained from an individual, adult male donor. Either horse Cyt *c* (2 × 10^–6^ M), human LRG1 (2 × 10^–8^ M, purified from human plasma), or both were added to parallel cultures at *t* = 0. The concentrations of Cyt *c* and LRG1 employed had yielded optimal effects in preliminary experiments. In the top panel cultured lymphocytes were examined for secondary necrosis by trypan blue exclusion and, in the bottom panel, for apoptosis by flow cytometry. Necrotic but not apoptotic cells absorb trypan blue. Apoptotic cells bind annexin V-FITC and do not bind propidium iodide and so are present in the lower right quadrants in the bottom panel (F.I., fluorescence intensity). The % apoptotic cells at day 7 were 20% in the control culture, 10% in the culture supplemented with LRG1, 36% in the culture supplemented with Cyt *c*, and 9% in the culture supplemented with both Cyt *c* and LRG1. [The figure was adapted from Codina et al. (2010) with permission from the publisher].

Having observed that LRG1 binds Cyt *c*, we sought to determine if LRG1 would have an impact on apoptosis in human and mouse lymphocytes induced by extracellular Cyt *c* (Codina et al., 2010). We hypothesized that due to steric hindrance LRG1 would have a protective effect. Results of a previously reported experiment using human lymphocytes obtained from one individual are shown in Figure 3. In the top panel secondary necrosis following a transition through apoptosis was monitored by trypan blue exclusion. Lymphocytes were cultured in fetal bovine serum-containing media from which bovine LRG1 had been depleted by adsorption through Cyt *c*-coupled Sepharose 4B.

The effect of the addition of Cyt *c* on cell viability in the cultures was most prominent in this case after day 7. Note the difference between untreated cells and cells incubated with Cyt *c* on day 10. LRG1 blocked the Cyt *c* effect (Codina et al., 2010). As previously reported, the drop-off in viability observed at day 7 for the subject’s lymphocytes shown in Figure 3 was more gradual and extended to day 10 in repeat analysis. The drop-off did not occur until day 10 for two other subjects’ lymphocytes and was more gradual for two others (Codina et al., 2010). The effect of Cyt *c* added to the cultures may not have been immediate in some cases due to the status of the lymphocytes at the time of harvest. Susceptibility to toxicity of extracellular Cyt *c* may require cells to enter a vulnerable state such as reduction in plasma membrane integrity possibly resulting from nutrient depletion over time.

The lower panel in Figure 3 shows results from flow cytometry where cells were labeled with propidium iodide (PI) and annexin V-FITC. Viable cells are not stained with these reagents. Apoptotic cells are labeled with annexin V-FITC only and appear in the lower right quadrant, while necrotic cells are labeled with both reagents and would appear in the upper right quadrant. At day 7, although distinctions in trypan blue exclusion were not apparent (Figure 3, top panel), apoptotic cells were clearly present in the culture treated with Cyt *c* but not so much in the other conditions tested. (Note again that apoptotic cells not yet advanced to the stage of secondary necrosis do exclude trypan blue.) By day 10 necrotic cells were observed in the cultures by flow cytometry (not shown; Codina et al., 2010).

**FIGURE 4:**
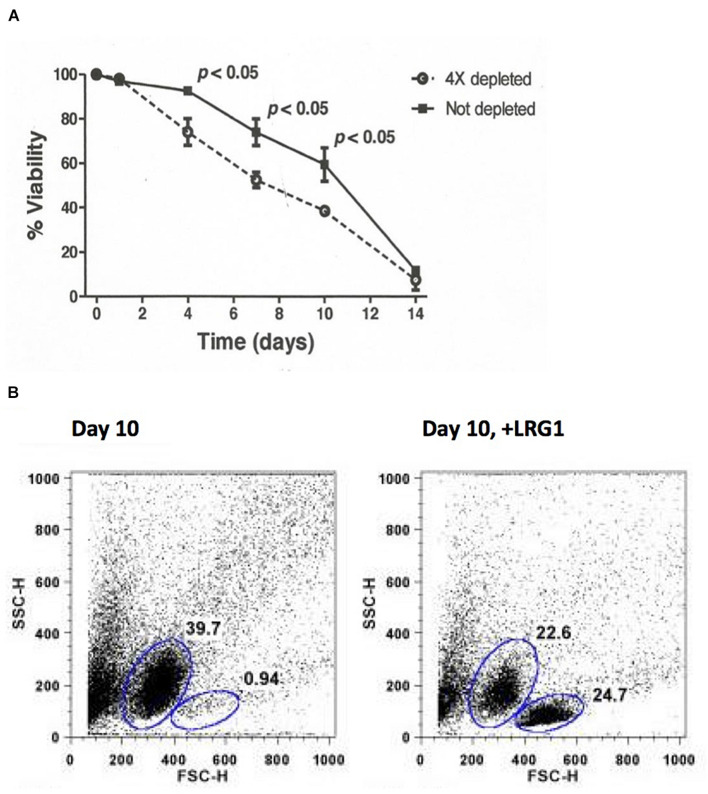
Depletion of LRG1 from culture media causes human lymphocytes to transition through an apoptotic phase faster than when LRG1 is present. **(A)** Depletion of LRG1 from culture media causes human lymphocytes to die faster. Dulbecco’s modified Eagle’s medium containing 10% fetal bovine serum was depleted of bovine LRG1 by adsorption through Cyt *c*-Sepharose 4B. Lymphocytes from three human subjects were cultured separately and viability was assessed by trypan blue exclusion. Averaged data were analyzed by two-sided Student’s *t*-test for paired comparisons. **(B)** Flow cytometry analysis (forward scatter versus side scatter) of cultured lymphocytes from one donor at day 10 showing 39.7% of the cells are apoptotic and only 0.94% are viable. Addition of human LRG1 has an anti-apoptotic effect (24.7% viable in this case) (adapted from Codina et al., 2010).

Consistently, exogenous LRG1 increased the viability of lymphocytes cultured without added Cyt *c* (Codina et al., 2010). When lymphocytes were cultured in media with or without depletion of LRG1, there was a statistically significant difference in survival (Figure 4, top). This shows that LRG1, a component of fetal bovine serum, contributes significantly to the viability of cultured lymphocytes. In the absence of LRG1 the lymphocytes transition through an apoptotic phase (Figure 4, bottom). Previously, we found that in LRG1-depleted media extracellular Cyt *c* was present in measurable amounts within 7 days in the cultures. It seems likely that the difference in lymphocyte viability between complete media and media depleted of LRG1 is the toxic effect of Cyt *c* released from dying cells that is not blocked due to LRG1 depletion. In support of this, antibodies specific for Cyt *c* had a similar protective effect as exogenous LRG1 in cultures to which Cyt *c* was not added (Codina et al., 2010).

Contrary to our expectation, the concentration of LRG1 that protected lymphocytes from Cyt *c*-induced apoptosis was 100-fold less than the concentration of Cyt *c* that was added to the cultures. This represents a vast molar excess of Cyt *c*. Thus, the protective effect of LRG1 did not appear to be simply due to steric hindrance. By comparing the abilities of human versus mouse LRG1 to protect lymphocytes obtained from both species we observed a species-preferential effect. Thus, while both human and mouse LRG1 protected human lymphocytes similarly from Cyt *c*-induced apoptosis, mouse LRG1 was more effective than human LRG1 in protecting mouse lymphocytes. This suggested that a component differing between mice and humans, in addition to LRG1, plays a role in transmitting the survival signal to lymphocytes. Furthermore, binding of LRG1 to Cyt *c* appeared to be required because LRG1 was not able to rescue cells from apoptosis induced by a Cyt *c* variant that LRG1 could not bind. Taken together these results suggest that LRG1 protected the cultured lymphocytes from apoptosis induced by extracellular Cyt *c* through a signaling mechanism involving a complex of Cyt *c* and LRG1. This was the first report proposing a biological function for LRG1 (Codina et al., 2010).

Extracellular Cyt *c* has been shown to upregulate the transcription factor nuclear factor kappa-light-chain-enhancer of activated B cells (NF-κB) in mouse splenocytes *in vitro* (Pullerits et al., 2005). In microglial cells, Cyt *c* was implicated in signaling through Toll-like receptor 4 (TLR4) which is known to result in NF-κB activation (Gouveia et al., 2017). NF-κB in lymphocytes is anti-apoptotic in some contexts (reviewed by Sen, 2006). In the studies by Pullerits et al. the culture medium was supplemented with fetal bovine serum that would have contained LRG1, so it is possible or, even likely, that upregulation of NF-κB could have been an effect of LRG1 complexed to Cyt *c*. The concentration of Cyt *c* most effective in activating the transcription factor was 10 μg/ml, with lesser activity observed at 100 μg/ml. The concentration of Cyt *c* employed in the study by Codina et al. cited above (25 μg/ml) is within this range. Conceivably then, the anti-apoptotic effect of the LRG1-Cyt *c* complex in that study could have involved activation of NF-κB. It is intriguing that TGF-β1 has the opposite effect in some contexts, inhibiting NF-κB activation resulting in apoptosis of B lymphocytes (Arsura et al., 1996). This finding involved culturing the lymphocytes in fetal bovine serum that likely contained LRG1, so it is possible that the complex of LRG1 and TGF-β1 was involved in the signal transduction leading to NF-κB inhibition.

Recently, we reported that intracellular LRG1 does protect against apoptosis induction in MCF-7 breast cancer cells in a steric manner by competing with Apaf-1 for binding Cyt *c* (Jemmerson et al., 2021; Table 1). LRG1 is synthesized as a 34 kD polypeptide and is glycosylated at five sites prior to secretion. The protein in blood is fully glycosylated, while the dominant form within cells is slightly lower in molecular weight (45 kD) due to partial glycosylation. Employing a monoclonal antibody reactive with non-glycosylated LRG1 we showed that partially glycosylated LRG1 enters the cytoplasm and binds Cyt *c* that may be released from mitochondria. The high affinity of LRG1 for Cyt *c*, much higher than the affinity of Apaf-1 for Cyt *c* (0.5 μM; Zhou et al., 2015), would make it an effective trap to sequester Cyt *c* and protect against apoptosis induction in non-committed cells. Cyt *c* was observed in the cytoplasm of viable MCF-7 cells transfected with the *LRG1* gene suggesting that when expression of LRG1 is increased in cells it may be a guardian against apoptosis induction when a few mitochondria undergo outer-membrane permeabilization in the absence of a committed apoptotic signal (so-called minority MOMP; Xu et al., 2020).

## Evidence Consistent With Leucine-Rich α_2_-Glycoprotein-1 and Cytochrome *c* Interaction in Lymphocyte Survival *in vivo*: Phenotypic Comparison of *Apaf-1* Knock-Out Mice, *K72A-Cyt c* Knock-In Mice, and *LRG1* Knock-Out Mice

Both Apaf-1 and LRG1 bind in the region around lysine 72 on Cyt *c*, as shown by their inability to bind tri-methyllysine 72 Cyt *c*, and compete for binding Cyt *c* (Kluck et al., 2000; Codina et al., 2010). If Apaf-1 was the only physiological ligand binding in the region around residue 72 on Cyt *c*, the phenotypes of *Apaf-1* knock-out and *K72A-Cyt c* knock-in mice (with a single mutation resulting in an amino acid substitution from lysine to alanine at position 72) should be identical in regard to apoptosis. Either the absence of Apaf-1, as in the *Apaf-1 knock-out* mice, or its inability to bind Cyt *c*, as in the *K72A-Cyt c* knock-in mice, should block Apaf-1-induced apoptosis. While these genetic variants often lead to gestational or perinatal lethality, in the mice that do survive the immunological phenotypes have striking differences (Yoshida et al., 1998; Hao et al., 2005; Table 2). Compared to *Apaf-1* knock-out mice, *K72A-Cyt c* knock-in mice have 100-fold fewer thymic cells, no pre-B cells detectable in the bone marrow, and 50-fold fewer splenic B and T lymphocytes. Thymocytes in *K72A-Cyt c* knock-in mice are markedly more sensitive to death stimuli than thymocytes from *Apaf-1* knock-out mice.

**TABLE 2:** Immunological phenotypes of genetically modified mice with defects in Cyt *c* interactions.

**Genetic modification**	**Immunological phenotype**	**References**
*Apaf-1* knock-out	Thymocyte development normal; other immune tissues not reported	Yoshida et al., 1998
*K72A Cyt c* knock-in	100-fold fewer thymocytes than *Apaf-1* knock-out mice that are more sensitive to death stimuli and 50-fold fewer splenic B and T lymphocytes	Hao et al., 2005
*LRG1* knock-out	Deficient in Th17 differentiation and lymphocytes in inguinal lymph nodes; other immune tissues not reported	Urushima et al., 2017

When cells from *K72A-Cyt c* knock-in mice were transferred into mice with the inability to recombine the genes required for expression of antigen-specific receptors on lymphocytes (*Rag-1* knock-out mice) and, hence, that cannot produce their own mature B and T lymphocytes, the lymphocyte populations that developed were comparable in number to normal controls. Thus, by transferring the *K72A-Cyt c* knock-in cells into a normal environment they developed normally indicating that the defect causing a diminution in lymphocyte numbers is “extrinsic” (Hao et al., 2005).

The unique phenotype of the *K72A-Cyt c* knock-in mice is consistent with the survival-promoting effect on both human and mouse lymphocytes that we observed for LRG1. In these mice extracellular LRG1 would not be able to bind variant Cyt *c* released from dying cells and, thus, could not promote survival. While Apaf-1 would not be able to induce apoptosis the extrinsic pathway of apoptosis would still be operative allowing Cyt *c* to be released from cells, not to mention other mechanisms that could cause Apaf-1-independent apoptosis (Imao and Nagata, 2013).

*LRG1* knock-out mice have been developed, although a thorough analysis of the immune system in these mice has not been reported to my knowledge. These mice are fertile with no outward phenotypic differences from normal mice (Wang et al., 2013). In a study of arthritis in these mice that was induced by collagen immunization, differentiation of Th17 cells, a subset of CD4^+^ T lymphocytes, was inhibited in *LRG1* knock-out mice and arthritis was dramatically attenuated (Urushima et al., 2017; Table 2). There was a significant decrease in lymphocyte numbers in inguinal lymph nodes, the only lymphoid tissue that was reported, although the levels of anti-collagen antibodies were similar in control and knock-out mice.

These results demonstrate a deficiency in the immune system of *LRG1* knock-out mice, the extent of which requires further examination. However, the immunological phenotype of the *LRG1* knock-out mice appears to be much less severe than what was observed in the *K72A-Cyt c* knock-in mice. As a number of potential binding partners of Cyt *c* have been identified (Martinez-Fabregas et al., 2014), it is conceivable that one or more of these binds in the region of residue 72 and plays additional roles in lymphocyte development. For example, translocation of Cyt *c* to the nucleus could enable molecular interactions that affect gene expression (Godoy et al., 2009). Any such interaction where residue 72 may be involved would still occur in the *LRG1* knock-out mice and would not occur in the *K72A-Cyt c* knock-in mice.

## Conclusion

Researchers from more than one laboratory have reported that LRG1 binds TGF-β1 (Saito et al., 2002; Takemoto et al., 2015) and that LRG1 binds Cyt *c* (Cummings et al., 2006; Shirai et al., 2010; Druhan et al., 2017). Here data are presented that LRG1 binds TGF-β1 and Cyt *c* simultaneously. This raises the possibility that the ternary complex serves as a signaling module. Some clues have emerged toward understanding the functional consequence. All three components have been shown to play roles in both cell death and survival. Cyt *c* is well documented as an inducer of apoptosis both inside (Liu et al., 1996) and outside cells (Ahlemeyer et al., 2002; Codina et al., 2010; Gouveia et al., 2017). When Cyt *c* is bound to LRG1 cell survival is promoted, at least for lymphocytes *in vitro*. LRG1 also affects both cell death (apoptosis) and survival (Table 1). Pending further study LRG1 appears to induce apoptosis through canonical TGF-β1 signaling and cell survival through non-canonical TGF-β1 signaling. A possible role for Cyt *c* in cell survival signaling should be considered.

The balance between death-promoting and survival effects of extracellular LRG1 could be influenced by the extent to which Cyt *c* is bound in the LRG1/TGF-β1 complex. Tethered together these three components may allow association of signaling polypeptides in the cell membrane that are not linked otherwise, thus altering the signals that are transmitted. Possible differences in the extent to which Cyt *c* is released during apoptosis among cell types and the accumulation of extracellular Cyt *c*, as well as expression of the appropriate receptor(s), could lead to variable effects of LRG1 on cell survival.

Cytochrome *c* is recognized as a damage-associated molecular pattern (DAMP; Eleftheriadis et al., 2016). In complex with LRG1 and under stress conditions, extracellular Cyt *c* could deliver a survival signal to cells in the microenvironment surrounding tissue damage to protect nearby cells not yet committed to apoptosis. Cyt *c* is increased in the blood of individuals with a variety of pathologies where apoptosis is implicated and so escapes phagocytic cell clearance in those conditions. It is not known whether Cyt *c* is released from apoptotic cells in healthy individuals. However, Cyt *c* is observed at low levels in the blood of many healthy human subjects and is released from apoptotic cells *in vitro* within 1–2 h of an apoptotic insult (Renz et al., 2001; Jemmerson et al., 2002; Eleftheriadis et al., 2016).

Apoptotic cell-derived extracellular vesicles escape phagocytosis and play various roles in normal physiology including cell survival, tissue regeneration, and differentiation (Li et al., 2020). Is it possible that Cyt *c* could be released in sufficient amounts from apoptotic cells as a consequence of regular “housekeeping,” such as from the billions of lymphocytes in primary immunological tissues that die daily, to play a role in these normal functions? Thorough analysis of the immunological phenotype of the *LRG1* knock-out mice would help determine whether LRG1 interactions play a key role in lymphocyte homeostasis.

The pleiotropic effects of TGF-β1 have been ascribed to the differentiation states of the cells that likely reflect the expression of receptor polypeptides and intracellular signaling molecules, other cytokines interacting with their receptors, and co-stimulatory molecules signaling pathways that complement activation of the requisite transcription factors (Li et al., 2006). Some of the pleiotropic effects of TGF-β1 that have been observed over the last several decades could be attributed to TGF-β1 in association with LRG1 rather than free TGF-β1. Likewise, effects of extracellular Cyt *c* that have been reported, such as activation of the transcription factor NF-κB in lymphocytes (Pullerits et al., 2005) and TLR4 engagement in microglial cells, upstream of NF-κB activation (Gouveia et al., 2017), could be due to Cyt *c* complexed to LRG1.

Depending on the context, TGF-β1 and NF-κB can be pro-apoptotic or anti-apoptotic factors (Lin et al., 1999; Sanchez-Capelo, 2005). TGF-β1 (possibly bound to LRG1) inhibits activation of the transcription factor NF-κB in B lymphocytes (Arsura et al., 1996). Inhibition of NF-κB induces apoptosis in some contexts (reviewed by Sen, 2006). Cyt *c* (possibly bound to LRG1) activates NF-κB and when bound to LRG1 inhibits or at least delays apoptosis in lymphocytes *in vitro*. Further research is needed to determine the extent to which LRG1 plays a role in the pleiotropic effects of TGF-β1 and whether the paradoxical effect of LRG1 on cell death and survival is due to inclusion of Cyt *c* in the complex with TGF-β1 and LRG1. If this turns out to be the case, the affects of one ligand on the other in terms of on-off rates could provide further insight into the signaling fate of responding cells. For example, when bound to LRG1, TGF-β1 could slow the on-rate for Cyt *c* and/or increase the off-rate, thus affecting the signaling kinetics and, potentially, the fate of a cell.

## Data Availability Statement

The raw data supporting the conclusions of this article will be made available by the author, without undue reservation.

## Ethics Statement

The studies involving human participants were reviewed and approved by the University of Minnesota Human Subjects Review. The patients/participants provided their written informed consent to participate in this study.

## Author Contributions

The author confirms being the sole contributor of this work and has approved it for publication.

## Conflict of Interest

The monoclonal antibody used to detect Cyt *c* in western blots was developed at the University of Minnesota and has been commercially licensed.

## Publisher’s Note

All claims expressed in this article are solely those of the authors and do not necessarily represent those of their affiliated organizations, or those of the publisher, the editors and the reviewers. Any product that may be evaluated in this article, or claim that may be made by its manufacturer, is not guaranteed or endorsed by the publisher.

## Abbreviations

Cyt*c*: cytochrome *c*
DAMP: damage-associated molecular pattern
EGFR: epidermal growth factor receptor
HUVECs: human umbilical vein endothelial cells
JAK: Janus kinase
LRG1: leucine-rich α _2_-glycoprotein-1
LRRs: leucine-rich repeats
Lgr4: LRR-containing G protein-coupled receptor 4
NF- κ B: nuclear factor kappa-light-chain-enhancer of activated B cells
STAT: signal transducers and activators of transcription
TGF- β 1: transforming growth factor-beta 1.
